# Late-onset epilepsy and subsequent increased risk of dementia

**DOI:** 10.18632/aging.202299

**Published:** 2021-01-10

**Authors:** Zhi-Ren Tsai, Han-Wei Zhang, Chun-Hung Tseng, Hsiao-Ching Peng, Victor C. Kok, Gao Ping Li, Chao A. Hsiung, Chun-Yi. Hsu

**Affiliations:** 1 Department of Computer Science and Information Engineering, Asia University, Taichung, Taiwan; 2 Department of Medical Research, China Medical University Hospital, China Medical University, Taichung, Taiwan; 3 Taichung City Smart Transportation Big Data Research Center, Taichung, Taiwan; 4 Pervasive Artificial Intelligence Research (PAIR) Labs, Hsinchu, Taiwan; 5 Biomdcare Corporation, New Taipei, Taiwan; 6 Program for Aging, China Medical University, Taichung, Taiwan; 7 Institute of Population Health Sciences, National Health Research Institutes, Miaoli, Taiwan; 8 Institute of Electrical Control Engineering, Department of Electrical and Computer Engineering, National Chiao Tung University, Hsinchu, Taiwan; 9 Department of Neurology, China Medical University Hospital, and School of Medicine, China Medical University, Taichung, Taiwan; 10 Disease Informatics Research Group, Asia University, Taichung, Taiwan; 11 Department of Internal Medicine, Kuang Tien General Hospital, Taichung, Taiwan; 12 Zhongshan Hospital, Affiliated Hospital of Fudan University, Shanghai, China; 13 Graduate Institute of Biomedical Science, China Medical University, Taichung, Taiwan

**Keywords:** epilepsy, dementia, Alzheimer’s disease

## Abstract

Inflammation is considered as a key pathogenesis factor of dementia and epilepsy. However, epilepsy’s association with dementia, particularly its role in the development of dementia, remains unclear. To evaluate the association between epilepsy and the risk of dementia, in Taiwan, we have now conducted a retrospective cohort study comprising 675 individuals (age, ≥50 years) with epilepsy and 2,025 matched control subjects without epilepsy. In order to match individuals diagnosed with epilepsy with those with no diagnosis of epilepsy (comparison cohort), we utilized exact matching at a ratio of 1:3. Compared with those in the comparison cohort, individuals in the epilepsy cohort had a significantly increased risk of developing dementia (adjusted hazard ratio = 2.87, *p* < 0.001). A similar result has been observed after stratifying for sex (adjusted hazard ratio in males = 2.95, *p* < 0.001; adjusted hazard ratio in females = 2.66, *p* < 0.001). To conclude, based on these data, epileptic individuals ≥50 years were at a greater risk of developing dementia than people who do not have epilepsy, which indicates that a diagnosis of epilepsy presents a greater risk for the development of dementia.

## INTRODUCTION

Dementia is one of the 15 top medical conditions that posed a significantly high disease burden over the last 10 years. Furthermore, together with epilepsy, it is one of the 25 top most common causes of life with disability worldwide [1–2]. Dementia is a progressive or long-lasting disease triggered by neurodegeneration and can be categorized according to the part of the brain affected and the rate of symptom worsening. Most common progressive diseases and conditions characterized by dementia include Alzheimer’s disease (AD) and vascular dementia (VaD) [3–4]. Among the etiologies of dementia in older individuals, the most frequent are AD and cerebrovascular disease [5]. Dementia secondary to AD is generally characterized by its subtle onset, whereas dementia onset in VaD is frequently found to be rapid. Nonetheless, both AD and VaD confer a progressive course that ultimately results in general cognitive deficiency and loss of functional independence [6–8].

On a global scale, AD is a frequent type of dementia [9] and accounts for more than 80% of the people >65 years of age with dementia [10]. Loss of memory, multiple cognitive deficiencies, and gradual deterioration of behavioral and functional capacities are among the characteristics of AD [11–15]. In most cases, AD pathology is associated with cerebral infarcts [16–17]. Besides AD, the most serious illness that leads to dementia is VaD, which comprises a group of different dementing diseases due to cerebrovascular insufficiency, and accounts for approximately 10% of dementia cases. In VaD, blood flow to the brain is reduced or blocked, causing decreased oxygen and nutrient supply to brain neurons, ultimately resulting in impairment of cognitive skills. Approximately 50% of patients with dementia display pathological characteristics of VaD [16–17].

Advances in medical technologies contribute to the increased aging population, and thus, the proportion of aged people with epilepsy is bound to escalate considerably worldwide. To date, multiple studies have investigated the potential risk factors for developing epilepsy in patients suffering from dementia and/or AD [18–24]. A previous study demonstrated that both dementia and AD posed nearly eight- and six-fold higher risk of epilepsy, respectively [19]. Another UK study showed that there is an association between significantly elevated risk of seizures and AD [20]. In Han Chinese patients, a higher risk of seizures was reported in AD patients than in age-matched controls [21]. Furthermore, the risk for developing epilepsy was found to be higher in AD patients with hyperlipidemia, and the risk was also high among male AD patients [22]. Additionally, the risk of epilepsy development is greater in patients with an AD diagnosis of >3 years [23], whereas the risk for the future development of epilepsy was observed to be higher in younger individuals with dementia [24].

However, some studies have demonstrated that cognitive dysfunction is more likely to affect elderly patients with epilepsy, and it was also suggested that an essential bidirectional relationship can occur between dementia and epilepsy. Therefore, some patients with epilepsy may be at a greater risk to develop dementia [25–27]. Several studies have examined the association between epilepsy and dementia. A previous study reported that patients with epilepsy, aged 50–75 years, were at a moderately high risk to develop dementia [27]. Another prospective case–control study showed an escalated risk for depression and undetermined disorders-like dementia, in adults with epilepsy and intellectual disability [28]. However, one study did not find any noticeable relationship between epilepsy and AD or dementia [29]. Furthermore, another case–control study did not observe a significant relationship between antiepileptic drugs use and dementia risk [30].

To summarize, the varied decline in the cognitive function in elderly people with epilepsy remains to be determined and would be an important matter for future studies. Longitudinal studies of elderly epileptic patients could be helpful [25]; furthermore, there were inadequate results to conduct a meta-analysis or pool the estimates from three different studies reporting the prevalent estimates for epileptic individuals in whom there was subsequent development of dementia, and neither of these studies evaluated the possible dementia risk factors [31]. Although several studies assessed the risk factors associated with epilepsy development in patients with dementia, the information on epidemiological data from the cases of patients with epilepsy, who developed dementia, was insufficient [31–32]. There are few epidemiological studies on the development of dementia in epileptic patients in Asia, and no studies have yet reported on the risk factors that associate with dementia problems in epilepsy. For instance, none of them have assessed sex, age, or clinical factors, such as the seizure or epilepsy severity, as potential risk factors for developing dementia. The association of epilepsy with the development of dementia and associated risk factors is not fully known. We aimed to conduct and compare a prospective, longitudinal study in a population-based cohort of patients with epilepsy focusing on their clinical and demographic characteristics and occurrence of dementia with that of control subjects who are non-epileptic, with a long-term follow-up. We used the data from the National Health Insurance Research Database (NHIRD) of Taiwan to evaluate the association of epilepsy with dementia and to establish if patients with epilepsy aged ≥50 years are at a greater risk of developing dementia than those without epilepsy in Taiwan.

## RESULTS

### Characteristics of the study population

After selecting patients and patient matching, the epilepsy and comparison cohorts comprised 675 and 2,025 individuals, respectively. Table 1 lists the demographic data and comorbid conditions of both cohorts. In the epilepsy cohort, the mean age was 62.5 ± 9.5 years, which was similar to the mean age of 62.4 ± 9.5 years in the comparison cohort. The individuals between 50 and 59 years of age comprised ~46% of the cohorts of patients with epilepsy and control subjects. For the parameters of many of the comorbidities, the analysis of matched patients revealed significant differences between the epilepsy and control cohorts. Expectedly, patients in the epilepsy cohort generally had more comorbidities than those in the comparison cohort.

**Table 1:** Characteristics of the epilepsy and comparison cohorts.

**Characteristics**	**n (%)**		***P* value**
	**Epilepsy cohort (n = 675)**	**Comparison cohort (n = 2,025)**	
**Age,** years			Matched
50**–**59	334 (49.5)	1,000 (49.4)	
60**–**69	167 (24.7)	496 (24.5)	
70**–**79	152 (22.5)	463 (22.9)	
≥80	22 (3.3)	66 (3.2)	
Mean ± SD	62.5 ± 9.5	62.4 ± 9.5	
**Sex**			Matched
Male	420 (62.2)	1,260 (62.2)	
**Comorbidities^a^**			
Hypertension	191 (28.3)	711 (35.1)	0.001
Diabetes mellitus	162 (24.0)	525 (25.9)	0.320
Coronary artery disease	194 (28.7)	492 (24.3)	0.022
Chronic obstructive pulmonary disease	196 (29.0)	352 (17.4)	<0.001
Hyperlipidemia	165 (24.4)	510 (25.2)	0.700
Cerebrovascular disease	313 (46.4)	386 (19.1)	<0.001
Head injury	69 (10.2)	113 (5.6)	<0.001
Depression	80 (11.9)	92 (4.5)	<0.001
Congestive heart failure	87 (12.9)	215 (10.6)	0.105
Atrial fibrillation	35 (5.2)	87 (4.3)	0.336
Cancer	137 (20.3)	333 (16.4)	0.022
Liver disease	191 (28.3)	345 (17.0)	<0.001
Chronic infection/inflammation	35 (5.2)	82 (4.0)	0.209
Autoimmune disease	6 (0.9)	5 (0.2)	0.023
Malnutrition	20 (3.0)	30 (1.5)	0.013
Parkinson’s disease	31 (4.6)	64 (3.2)	0.080

SD, standard deviation.

Values are expressed as means ± SD or number (percentage).

^*a*^Comorbidities were defined during the follow-up period.

### Associations between epilepsy and dementia

Table 2 lists the associations of dementia risks between the cohorts of patients with epilepsy and control subjects. The incidence rate of dementia was 12.37 per 1,000 person-years in the epilepsy cohort and 4.65 per 1,000 person-years in the comparison cohort. In the epilepsy cohort, the crude hazard ratio (HR) for dementia (2.66; 95% confidence interval [CI], 1.96–3.59; *p* < 0.001) was significantly higher than that in the comparison cohort; moreover, the epilepsy cohort had a significantly higher adjusted HR for dementia (2.87; 95% CI, 2.07–3.99; *p* < 0.001) after correcting for possible confounding due to the demographic variables and comorbidities. These results imply that, among individuals aged ≥50 years with epilepsy, the possibility of having a new diagnosis of dementia is 187% more than in those without epilepsy. Furthermore, the adjusted HRs (95% CIs) for dementia in the epilepsy cohorts were 2.95 (1.90–4.57) for males and 2.66 (1.62–4.37) for females. After stratifying data according to sex, both sexes showed statistically significant correlations between dementia and the epilepsy cohorts compared with those in the comparison cohorts.

**Table 2:** Dementia incidence and HRs for dementia in the epilepsy and comparison groups.

Population*	**Study group**	**Dementia**	**PY**	**Rate^*a*^**	**Crude HR (95% CI)**	**Adjusted HR^*b*^ (95% CI)**
Total	Comparison (n = 2,025)	96	20,627	4.65	1 (reference)	1 (reference)
	Epilepsy (n = 675)	75	6,062	12.37	2.66 (1.96–3.59)^‡^	2.87 (2.07–3.99)^‡^
Male	Comparison (n = 1,260)	54	12,513	4.32	1 (reference)	1 (reference)
	Epilepsy (n = 420)	46	3,642	12.63	2.94 (1.98–4.36)^‡^	2.95 (1.90–4.57)^‡^
Female	Comparison (n = 765)	42	8,114	5.18	1 (reference)	1 (reference)
	Epilepsy (n = 255)	29	2,420	11.98	2.30 (1.44–3.70)^‡^	2.66 (1.62–4.37)^‡^

CI, confidence interval; HR, hazard ratio; PY, person-years; Rate, incidence rate.

^*a*^per 1,000 person-years.

^*b*^Cox regression models were corrected for age, sex, head injury, coronary artery disease, cancer, chronic obstructive pulmonary disease, hypertension, cerebrovascular disease, depression, liver disease, malnutrition, and autoimmune disease.

^‡^*p* < 0.001.

*stratified by sex.

**Figure 1:**
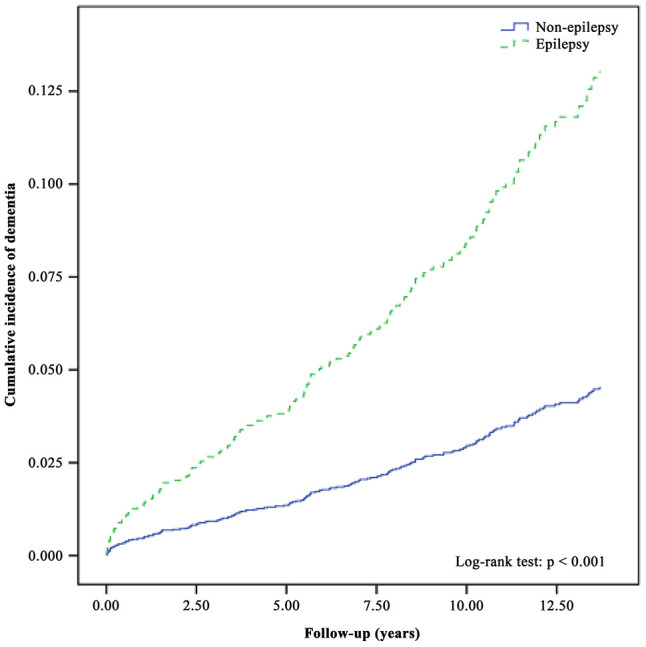
**The cumulative incidence curves of dementia in individuals with and without epilepsy.**

During the follow-up period of 13 years, the cumulative dementia incidence curves in the epilepsy and comparison cohorts, which were evaluated using the Kaplan–Meier method (Figure 1), showed that the incidence rate was significantly higher in the epilepsy cohort than that in the control cohort (log-rank test, *p* < 0.001).

### Risk of dementia and monthly frequency of hospital visits for epilepsy

Table 3 lists the association between changes in the monthly hospital visits frequency for epilepsy and dementia risk. Based on the frequency of outpatient/inpatient hospital visits per month for epilepsy, patients with epilepsy were separated into two groups. The dementia incidence rates were, respectively, 9.22 and 39.15 per 1,000 person-years among patients with <1 and ≥1 hospital visit per month for epilepsy. Following corrections for sex, age, and unbalanced comorbidities, in comparison with those in the cohort control, individuals in the epilepsy cohort with <1 and ≥1 hospital visits per month for epilepsy had a 2.07-fold (95% CI, 1.44–2.98; *p* < 0.001) and 11.20-fold (95% CI, 7.02–17.86; *p* < 0.001) risk of developing dementia, respectively. When more frequencies of outpatient/hospitalization per month for epilepsy were visited, patients had a higher risk of developing dementia (*p* for trend <0.001).

**Table 3:** HRs for dementia according to the frequency of hospital visits per month for epilepsy.

**Frequency of hospital visits per month for epilepsy**	**Dementia**	**PY**	**Rate^*a*^**	**Adjusted HR^*b*^ (95% CI)**
Comparison cohort	96	20,627	4.65	1 (reference)
<1	50	5,424	9.22	2.07 (1.44–2.98)^‡^
≥1	25	639	39.15	11.20 (7.02–17.86)^‡^
*p* for trend				<0.001

CI, confidence interval; HR, hazard ratio; PY, person-years; Rate, incidence rate.

^*a*^per 1,000 person-years.

^*b*^Cox regression models were corrected for age, sex, coronary artery disease, depression, chronic obstructive pulmonary disease, cancer, cerebrovascular disease, hypertension, head injury, liver disease, malnutrition, and autoimmune disease.

^‡^*p* < 0.001.

## DISCUSSION

This population-based cohort study, conducted in Taiwan, in which the data on national insurance claims have been applied to investigate the association between epilepsy and dementia, reported that individuals aged ≥50 years with epilepsy were at a greater risk to develop dementia and that the development of dementia is more likely to occur among male patients with epilepsy. During the 13-year longitudinal follow-up period, the progression of dementia escalated with the increasing occurrence of epilepsy. Furthermore, in the present study, we demonstrated that the occurrence of dementia in patients with epilepsy with ≥1 outpatient visit and/or hospitalizations per month was significantly more than in those in the comparison cohort.

A much accumulating clinical evidence provides strong support to the essential role of inflammation in the pathophysiology of epilepsy in humans. Studies on resected brain tissue samples obtained from patients with epilepsy undergoing brain surgery reported the occurrence of a complex and persistent evidence for inflammation, such as the activation of astrocytes and microglia and the formation of molecules that are proinflammatory [33]. Experimental data from numerous studies indicated that epilepsy/seizures could trigger a persistent inflammatory reaction and lead to neuronal damage and death [34–35]. Immunohistochemical studies on experimental epilepsy models demonstrated that different molecules that cause inflammation were induced rapidly by brain injury or seizures via microglia and astrocytes that are locally activated, indicating that the initial inflammatory reaction, due to any brain damage-causing process, occurs in the parenchymal brain cells [36–38]. Furthermore, experimental studies have demonstrated the significant role of glial cells, which can be activated in the brain by different injuries, in the pathogenic processes of precipitation and recurrence of epilepsy/seizure [39]. Specifically, either injury to the brain or proconvulsant events can cause the activation of astrocytes and microglia and induce the release of several types of inflammation-causing molecules from these cells, thereby triggering a cascade of inflammatory pathways in the brain [39]. Proinflammatory mediators can modify neuronal excitability and derange the normal physiological roles of glia either by autocrine or paracrine mechanisms, thereby disrupting glioneuronal communication [39]. Epilepsy/seizures can trigger a substantial inflammatory reaction in parenchymal cells, including microglia, as well as neurons, e.g., the expression levels of NF-κB and cyclo-oxygenase 2 are significantly increased in neurons after a seizure [40]. The inflammation induced by epilepsy/seizures was due to intricate neurophysiological events that are unique to the brain and varied in duration and participating cell populations from typical immune responses induced by bacterial/viral infections [40]. It is worth noting that epilepsy/seizures can cause continuous neural reorganization and contribute to progressive worsening of epilepsy and the cognitive and behavioral consequences. Furthermore, in animal studies, the decline in spatial memory in rats is often associated with an increase in the number of seizures kindled [35]. The results of neuroimaging studies indicate that patients with refractory (i.e., drug-resistant) temporal lobe epilepsy may be at risk of progressive structural and associated cognitive impairment. However, both seizure-induced cognitive impairment and the severity of epileptic seizures are influenced by the extent of damage caused by seizures [41].

In the present study, we revealed that individuals with epilepsy were at a higher risk to develop dementia. We hypothesized that the possible mechanisms underlying this association are inflammatory processes in the brain, which were triggered by an insult that is epileptogenic and may contribute to the development of dementia. In a previous study, it has been proposed that seizures that occur in the temporal lobe could damage the hippocampus over time, resulting in progressive memory loss [42]. Notably, chronic inflammation is associated with a broad spectrum of neurodegenerative diseases associated with aging, including AD [43]. Recently, we have shown that chronic exposure to hydrocarbons in the ambient air was correlated with the ischemic stroke and dementia risk [44–45]. Several studies have reported on the presence of inflammatory markers, including activated microglia and inflammatory proteins, in the brains of AD patients as evidence for the contribution of neuroinflammation to AD pathogenesis [46–56]. Neuroinflammation can play a critical role in the pathogenesis of dementia by inducing neuronal damage through inflammatory mechanisms. A proposed mechanism attributes a causative role to amyloid beta-mediated activation of an inflammatory cascade, which leads to direct amyloid beta-mediated neurotoxicity in AD [57]. It is proposed that microglial cells, inflammatory cytokines, and complement proteins contribute to neurodegeneration and may participate in neuronal loss in AD as primary role players rather than as part of phenomena secondary to the primary degenerative processes [57]. Moreover, it has been proposed that strong inflammatory responses to viral infections were responsible for the increased risk of AD. These results firmly suggest that inflammatory responses in the brain, such as those caused by a viral infection, initiate and/or accelerate the pathological processes underlying AD [58].

Presently, animal and human models indicate the significance of various pathophysiological events during the formation of epileptic seizures as well as the manifestation of dementia, such as the deranged function of the cytoskeleton. These changes contribute to disturbed neuronal structure and compromised neurotransmitter systems [59], elevated expression of amyloid-β protein, altered cerebrovascular system, neuronal hyper synchronization, and synaptic depression, as well as neurotrophic factor signaling or oxidative stress [60–63]. In the mouse model of Dravet syndrome (a damaging epileptic encephalopathy), the deletion of tau gene alleles reduced the incidences of seizures and improved learning and memory [64]. The administration of sodium selenate, a chemical that decreases hyperphosphorylation of tau, reduced the frequency and severity of seizures in three distinct rodent models of epilepsy [65]. Among the underlying pathological pathways that are common to epilepsy and dementia, it was shown that whereas suppression of hyperphosphorylation of tau benefits epilepsy, reduction in seizures is helpful in AD [25].

The present study has some limitations, even though there was a considerable benefit from the large sample size obtained from the Taiwan NHIRD. First, biases that may arise from unidentified confounders may have affected the results, and there were no clinical risk factors for dementia (e.g., blood pressure, blood sugar levels, and levels of lipoproteins) available in the Taiwan NHIRD. Second, since relevant laboratory data could not be obtained from the NHIRD, the severity of epilepsy could not be clearly categorized in the present study. Third, extrinsic factors affecting dementia, including toxic environmental factors at and during daily living activities, which could enhance inflammation, could not be obtained from the NHIRD.

## CONCLUSION

The present study revealed that patients with epilepsy aged ≥50 years have a higher risk and incidence of dementia than the comparison cohort without epilepsy in Taiwan, which indicates that epilepsy is associated with elevated dementia risk. In the sex-stratified analysis, both males and females in the epilepsy cohort were at a greater risk of the development of dementia than those in the comparison cohort. Furthermore, male patients with epilepsy were more likely to develop dementia.

## MATERIALS AND METHODS

### Data source

The data that include the claims data for one million randomly sampled beneficiaries during 1996–2013 were procured from the Longitudinal Health Insurance Database (LHID) within the NHIRD in Taiwan. The period of observation was fixed as 2000–2013 to enhance the reliability of the NHIRD data. The Taiwan Ministry of Health and Welfare Clinical Trial Center (MOHW109-TDU-B-212-114004) partly approved the present study. The Research Ethics Committee waived the right of attaining informed consent because deidentified/anonymized data were utilized from the database. All results produced or analyzed as part of this study are incorporated in this article.

The NHIRD, established in 1996 in Taiwan, contains all the medical histories and insurance claims data obtained from the National Health Institute (NHI) database that contains the healthcare information of 22.96 million people (99% of the population of Taiwan), covered by a universal health insurance program [66]. The NHIRD holds data on the outcomes of real-world practice and has been mostly recognized for its significance and clinical influence beyond the results obtained from clinical trials conducted at multiple centers that provide support for developing guidelines for disease management in clinical practice [67]. The NHI has instituted firm rules for record keeping to identify fraud and misconduct in medical practice and can levy penalties that are 100 times the healthcare expenditure claims to prevent any medical frauds. Hence, the NHIRD delivers high-quality information on healthcare, which is reliable and honest for investigating real-world evidence, that is based on big health data analytics. The database records the health status of all individuals applying the International Classification of Disease, 9^th^ Revision, Clinical Modification (ICD-9-CM).

### Study design and population

**Figure 2:**
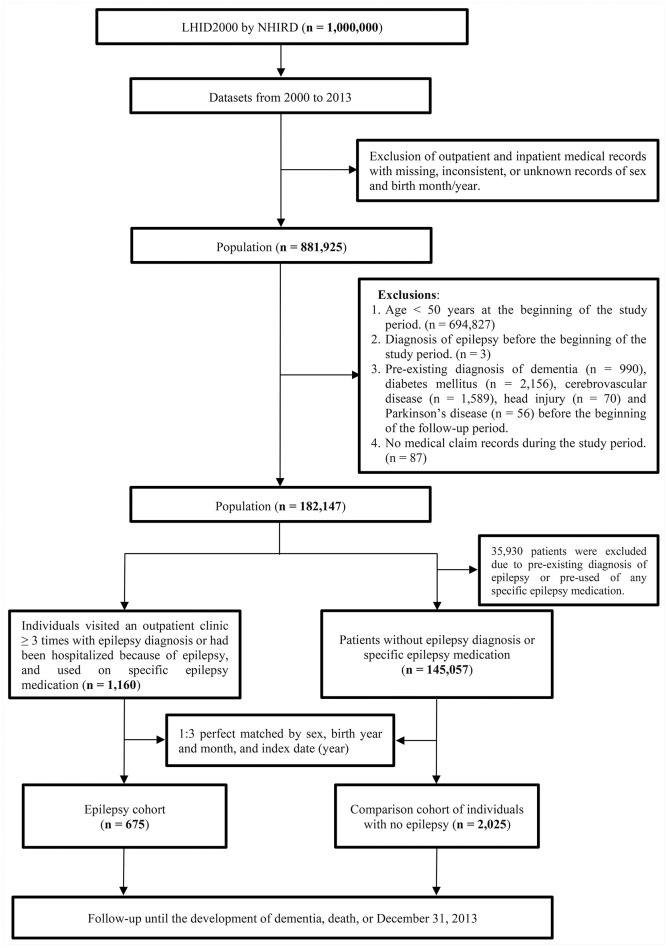
**The flow of the study population.**

This retrospective cohort study encompassing the period between January 1, 2000, and December 31, 2013, included the inpatient and outpatient claim data from the LHID. Among the one million beneficiaries in the LHID, we excluded those with lost/unidentified records for sex and birth month/year. After excluding individuals aged <50 years, those aged ≥50 years at the start of the study duration (2000–2013) were enrolled (n = 187,098). Additionally, we excluded individuals diagnosed with epilepsy before the start of the study duration (n = 3); those with dementia occurrence before the beginning of the follow-up period (n = 990); and those with pre-existing diagnoses of diabetes mellitus (n = 2,156), cerebrovascular disease (n = 1,589), head injury (n = 70), and Parkinson’s disease (n = 56) before the start of the follow-up period. Moreover, individuals with no medical claim records during the follow-up period (n = 87) were excluded. Ultimately, we included 182,147 individuals for the present study. Figure 2 shows the selection flow of the study population.

### Selection and measurement of exposure

From the included population, we determined individuals diagnosed with epilepsy between 2000 and 2013 based on the ICD-9-CM code 345 who were treated with specific epilepsy medications such as phenytoin, carbamazepine, primidone, valproic acid, phenobarbital, and gabapentin. Individuals who reported having visited an outpatient clinic at least three times or more with an epilepsy diagnosis or had been hospitalized because of epilepsy (based on the ICD-9-CM code 345) throughout the study period were included in the epilepsy cohort. The comparison cohort comprised individuals from the same dataset with no diagnosis of epilepsy and who were not treated with specific epilepsy medications. This cohort was established using exact matching at a ratio of 1:3 according to birth year and month, sex, and the index year. Eventually, 2,700 individuals were recruited for the two cohorts. Of these, 675 individuals were included in the epilepsy cohort, and 2,025 individuals were included in the control cohort. For all subsequent analyses, the very first date of hospitalization or visit to the outpatient clinic associated with a diagnosis of epilepsy and the first claim record date in the study period were defined as the index date for the epilepsy cohort and the control cohort, respectively; the study individuals were followed from then until 2013.

### Definition of incidence of dementia

For the epilepsy and comparison cohorts, the dementia incidence rate was expressed as the number of events per 1,000 person-years. In the present study, the primary outcome measure was new cases of dementia diagnosis throughout the period of the study based on ICD-9-CM codes 290, 294.1, and 331.0 defined in at least three separate medical claims released in an outpatient situation (to minimize accidental miscoding in the outpatient reimbursement data) or in one claim issued in an inpatient setting. The very first hospitalization or an outpatient clinic visitation date, with dementia diagnosis, was defined as the date of diagnosis and also regarded as the date of recently diagnosed dementia for all successive analyses. We used dementia diagnosis, death, or December 31, 2013, of the final date of observation, whichever occurred first, as the endpoint from the index date.

### Comorbidities

Information on the associated disease conditions of the individuals was evaluated based on ICD-9-CM codes in the LHID. Comorbidities that were considered in the analyses were as follows: coronary artery disease (410–414), hypertension (401–405), cerebrovascular disease (430–438), chronic obstructive pulmonary disease (490–492, 494, and 496), diabetes mellitus (250), hyperlipidemia (272), head injury (310.2, 800, 801, 803, 804, 850, 851, 853, 854, and 959.01), congestive heart failure (428), depression (296.2, 296.3, 296.82, 300.4, and 311), atrial fibrillation (427.31 and 427.3), liver disease (571 and 070), cancer (140–165, 170–176, and 179–208), chronic infection/inflammation (042, 010–018, and 090–099), malnutrition (260–269) autoimmune disease (279), and Parkinson’s disease (332). These comorbid conditions were recognized and described according to the history of diagnosis acquired from a minimum of three outpatient visits or at least one hospitalization before the date of newly diagnosed dementia in order to minimize the influence of bias due to data selection in the study database.

Studies have shown that people with epilepsy have increased frequency of most chronic conditions compared with that in the general population. Such conditions include high blood pressure, stroke, asthma, migraine, chronic bronchitis, and cancer [68]. Additionally, in comparison with the general population, epilepsy has been shown to be associated with a greater occurrence of mental health disorders, particularly, depression and anxiety [69]. Epilepsy is triggered by numerous factors and recognized by a high rate of discharge of specific neurons [70]. Comorbidities may directly lead to epilepsy. Cerebrovascular diseases, such as stroke, are regarded as the most frequent reason for epilepsy in older people [71–73]. Several studies in experimental models and humans have shown that the changes observed after stroke included critically decreased regional blood flow, a low regional cerebral oxygen metabolic rate, and increased blood–brain barrier permeability, which make the brain prone to seizures/epilepsy [74–76].

The damage to the brain due to elevated blood pressure could decrease the threshold for seizure and thus trigger epilepsy [77]. Instead, the onset of seizure is related to amplified sympathetic tone, which might by itself lead to higher blood pressure [78]. Chronic obstructive pulmonary disease that comprises emphysema and chronic bronchitis is a frequent somatic comorbidity in patients with epilepsy. A study found a higher occurrence of chronic obstructive pulmonary disease in a US adult population with epilepsy [79]. Epilepsy and seizures are among the probable effects of traumatic brain injury. Traumatic brain injury can lead to several changes that are possibly epileptogenic, which include vascular, axonal and neuronal damage, and subarachnoid and parenchymal hemorrhage. Traumatic brain injury triggers cascades of changes at molecular and cellular levels, including gliosis, excitotoxicity, and neuroinflammation, as well as later toxicity due to iron-rich breakdown products of hemoglobin [80–83]. Different kinds of brain tumors can also cause epilepsy. Among all types of brain cancers, glioneuronal tumors, such as gangliogliomas and dysembryo-plastic neuroepithelial tumors, are quite likely to present seizures as the symptom [84–87].

A study reported that a deterioration of depression may enhance the risk of developing epilepsy and also increase the odds of severe seizure outcomes. Frequent underlying pathophysiological mechanisms can elucidate the risk of developing epilepsy after incident depression [88]. Several studies have suggested that inflammatory mechanisms in the brain that comprise innate immunity machinery play a crucial part in the pathophysiology of epilepsy [36, 89]. People with autoimmune disease also appear to be at increased risk of developing epilepsy compared with those in the general population [90]. Furthermore, epilepsy has not been regarded to be associated with Parkinson's disease [91]. Nevertheless, a study indicated that the coexistence of Parkinson's disease and epilepsy may lead to difficulties in diagnosing or correctly interpreting the etiology of paroxysmal neurological episodes and the possible modifying effect that Parkinson's disease and epilepsy may have on each other [92]. Adjustments for significantly unbalanced comorbidities were conducted in the present analyses to avoid potential bias due to these comorbidities.

### Statistical analyses

The descriptive data were presented as mean ± standard deviation (SD) and frequencies with percentages (%) for continuous and categorical variables, respectively. The chi-squared test and Student’s ***t*** test were applied to evaluate the differences in demographic characteristics and distribution of comorbidities between the matched control and epilepsy cohorts. The dementia risk between the cohorts, expressed as HRs with 95% CIs, was evaluated with the Cox proportional hazards regression model. We estimated the independent effects of epilepsy in incident dementia by adjusting for age, sex, and comorbidities with unbalanced distribution and examined whether the effects differed between males and females in stratified analysis. Furthermore, we attempted to determine the risk of dementia concerning the frequency of outpatient/inpatient hospital visits per month because of epilepsy compared with that in the comparison cohort. To elucidate the relationship between the presence of epilepsy and the development of dementia, we conducted the Kaplan–Meier analysis to ascertain the possibility of individuals with dementia during the follow-up period; the log-rank test was applied to evaluate the difference between the two cohorts. All analyses were conducted with CareStore X1 Studio Research Platform and the Statistical Product and Service Solutions (SPSS; Version 22). All statistical tests were two-sided and differences were considered to be statistically significant when the *p* values are ≤0.05.
